# Dexmedetomidine for the prevention of postoperative delirium in elderly patients undergoing noncardiac surgery: A meta-analysis of randomized controlled trials

**DOI:** 10.1371/journal.pone.0218088

**Published:** 2019-08-16

**Authors:** Hai Zeng, Zunjiang Li, Jianbin He, Wenbin Fu

**Affiliations:** 1 The Second Clinical College, Guangzhou University of Chinese Medicine, Guangzhou, Guangdong Province, China; 2 School of Basic Medicine, Guangzhou University of Chinese Medicine, Guangzhou, Guangdong Province, China; 3 Department of Acupuncture and Moxibustion, The Second Affiliated hospital of Guangzhou University of Chinese Medicine (Guangdong Provincial Hospital of Chinese Medicine), Guangzhou, Guangdong Province, China; 4 Shenzhen Bao’an Research Center for Acupuncture and Moxibustion, Shenzhen, Guangdong Province, China; 5 Sanming Project of Medicine in Shenzhen (No. SZSM201806077), Shenzhen, Guangdong Province, China; University of Mississippi Medical Center, UNITED STATES

## Abstract

**Background:**

Postoperative delirium (POD) among the elderly population that undergoes noncardiac surgery is significantly associated with adverse clinical outcomes. We conducted this meta-analysis to evaluate the effectiveness and safety of dexmedetomidine for the prophylaxis of POD among the elderly population after noncardiac surgery.

**Methods:**

We searched Embase, PubMed, and the Cochrane Library from inception date to March 2019 for randomized controlled trials (RCTs) that compared dexmedetomidine and placebo for the prevention of POD and evaluated the major cardiovascular outcomes among elderly people after noncardiac surgery. Two authors independently screened the studies and extracted data from the published articles. The main outcome was the incidence of POD. The secondary outcomes included the occurrence of bradycardia, hypotension, hypertension, tachycardia, myocardial infarction, stroke, hypoxaemia, and all-cause mortality.

**Results:**

A total of 6 RCTs with 2102 participants were included. Compared with placebo, dexmedetomidine significantly reduced the prevalence of POD (RR = 0.61, 95% CI 0.34–0.76, *P* = 0.001, *I*^*2*^ = 66%), and the risk of tachycardia (RR = 0.48, 95% CI 0.30–0.76, *P* = 0.002, *I*^*2*^ = 0%), hypertension (RR = 0.59, 95% CI 0.44–0.79, *P* < 0.001, *I*^*2*^ = 20%), stroke (RR = 0.22, 95% CI 0.06–0.76, *P* = 0.02, *I*^*2*^ = 0%), and hypoxaemia (RR = 0.50, 95% CI 0.32–0.78, *P* = 0.002, *I*^*2*^ = 0%) in elderly patients who underwent noncardiac surgery. However, dexmedetomidine accelerated the occurrence of bradycardia (RR = 1.36, 95% CI 1.11–1.67, *P* = 0.003, *I*^*2*^ = 0%). Furthermore, no significant differences were observed in the incidence of hypotension, myocardial infarction, and all-cause mortality between the dexmedetomidine and placebo groups.

**Conclusions:**

Among elderly patients after noncardiac surgery, the prophylactic use of dexmedetomidine, compared with the use of placebo, was related to a decline in the incidence of POD.

## Introduction

Delirium is usually characterized as a multifactorial syndrome of acute attention and cognitive disorders; delirium is also a common, serious, underrecognized, and even lethal condition, especially for geriatric patients [1]. Delirium is associated with an elevated risk of mortality, complication morbidity, and dementia, an extended length of hospital stay, and a worsening in health-related quality of life [2–6]. The prevalence of postoperative delirium (POD) in elderly patients after noncardiac surgery is approximately 13% to 50% [1]. To decrease the incidence and adverse outcomes associated with delirium, multicomponent nonpharmacologic approaches that help control multiple risk factors of delirium are recommended [7, 8]. However, to date, the use of pharmacologic intervention to prevent delirium remains controversial [1, 8–11].

Dexmedetomidine serves as a potent sedative and has positive sedation and analgesic effects with modest anxiolytic ability, minimal respiratory depression and neurotoxicity; the mechanism of dexmedetomidine is closely related to its highly selective stimulation of the alpha-2 adrenoreceptors [12, 13]. In recent years, the usage of dexmedetomidine has increased for critically ill patients, particularly for surgical patients [14].

Two published meta-analyses [15, 16] showed that dexmedetomidine might decrease the occurrence of POD, compared with other active sedative drugs, in adult patients who underwent cardiac surgery. However, it is not clear whether dexmedetomidine exerts benefits on delirium prevention after noncardiac surgery among the elderly population when the drug is compared with placebo. Recent randomized controlled trials (RCTs) have reported inconsistent conclusions about the prophylactic effect of dexmedetomidine on POD in elderly patients. As new studies continue to provide extensive new data and insights into the potential effects of dexmedetomidine on POD and other major clinical outcomes, this review was performed to estimate the updated influence of dexmedetomidine on POD by comparing the drug with placebo in elderly patients after noncardiac surgery.

## Materials and methods

The meta-analysis was reported in accordance with the Preferred Reporting Items for Systematic Reviews and Meta-analysis (PRISMA) criteria [17]. The protocol of our review was registered in the International Prospective Register of Systematic Reviews (PROSPERO) (registration no. CRD42018105508).

### Search strategy

Two independent reviewers conducted systematic literature searches. The searches included PubMed, Embase, and the Cochrane Library databases. The literature search of each database was up to date as of March 2019. A language restriction on studies published in English was imposed. The study design was limited to RCTs. The following Medical Subject Headings (MeSH) or non-MeSH terms and their combinations were searched in the title and abstract: “dexmedetomidine”, “precedex”, “placebo”, “saline”, “Sodium Chloride”, “salt water”, “placebos”, “elder”, “aged”, “elderly”, “geriatric”, “old”, “older”, “elders”, “geriatrics”, “intraoperative”, “surgery”, “surgical”, “operation”, “postoperative”, “anesthesia”, “anaesthesia”, “operative”, “perioperative”, “clinical”, “trial”, and “random”. The complete search strategies of PubMed and Embase are presented in the supplementary (S1 File). Any disagreements reached a consensus by discussing with a third reviewer.

### Inclusion criteria

Patients: elderly population (aged 65 years or older) who underwent noncardiac surgery under general anesthesia; intervention: dexmedetomidine; comparison: placebo (normal saline); primary outcome: the incidence of POD; and secondary outcomes: the occurrence of major cardiovascular events (bradycardia, hypotension, hypertension, tachycardia, stroke, and myocardial infarction), hypoxaemia, and all-cause death. The study design was limited to RCTs.

### Exclusion criteria

Duplicated reports, trials that did not report the outcomes of interest, and case reports were excluded. We also eliminated trials in which other interventions were conducted in addition to the use of dexmedetomidine (e.g., nonpharmacological interventions and use of antipsychotics).

### Data extraction

Any selected study that fulfilled the inclusion criteria was included. The data from each eligible study were extracted by two independent reviewers. Discrepancies were reconciled after discussing with a third reviewer. The data extracted included the year of publication, the first author’s name, basic characteristics of the participants (average age, sex ratio, and sample size), countries where the trials were conducted, surgery and anesthesia type, assessment method and time of POD, intervention time of dexmedetomidine, the sedative dose and rate of dexmedetomidine infusion, and the primary and secondary outcomes mentioned above.

### Quality assessment

Using the Cochrane Collaboration’s tool for evaluating the risk of bias, the quality of each included trial was independently estimated by two investigators, with any discrepancies reconciled by a third reviewer. The tool had 7 quality items: random sequence generation, allocation concealment, blinding of participants and personnel, blinding of outcome assessment, incomplete outcome data, selective reporting, and other bias [18]. Each item was classified as a low, unclear, or high risk of bias [18].

### Data analysis

Statistical analyses were completed with RevMan (version 5.3.5) and Stata (version 14.0) software. For dichotomous outcomes, summary relative risks (RRs) with corresponding 95% CIs were calculated. A *P* value of less than 0.05 was deemed statistically significant. Heterogeneity was calculated quantitatively using the *I*^*2*^ statistic. The *I*^*2*^ statistic was deemed to represent a no (0%), low (0%-25%), moderate (25%-75%), and high (75%-100%) likelihood of heterogeneity [19]. A random-effects model was adopted since there was heterogeneity among studies. We used the Egger regression test and the Begg’s rank correlation test to examine the publication bias in addition to visual measurements of the funnel plots.

## Results

### Search results

**Fig 1:**
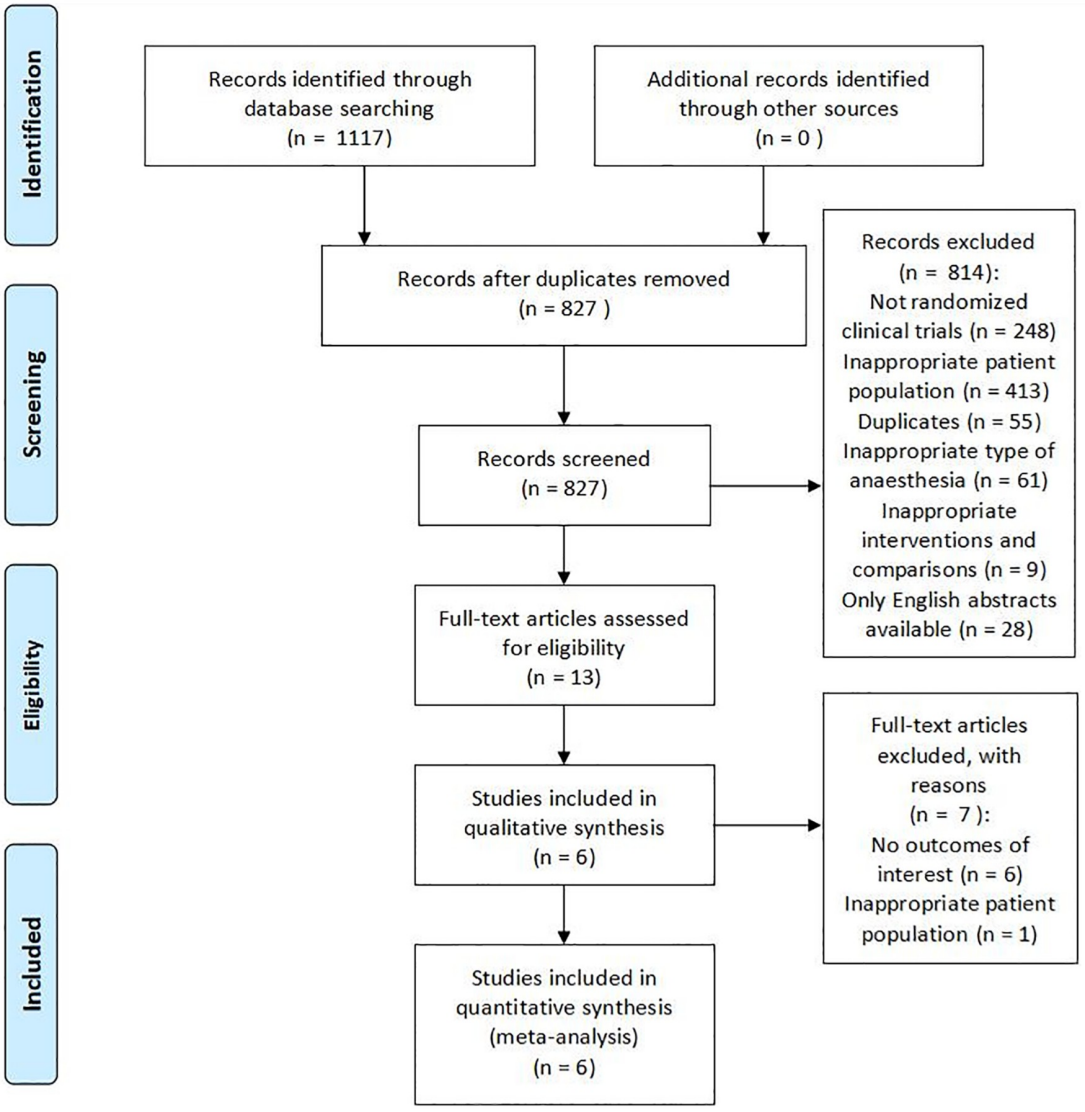
PRISMA flow diagram.

The initial literature search yielded 1117 studies. The number of duplicated articles removed was 290. A total of 813 studies were excluded after screening the titles and abstracts. Thirteen studies were reviewed for eligibility by scrutinizing full-text articles. Of those studies, 6 studies [20–25] reported no relevant clinical outcomes of interest, and one study [26] involved participants who were younger than 65 years. Finally, 6 eligible RCTs (2102 elderly patients) [27–32] were included in our review. The PRISMA flowchart is presented in Fig 1. Additionally, the interrater reliability measures between the two reviewers showed high agreement with kappa values of 0.83 for systematic searches and 0.89 for study selection.

### Study characteristics

The basic characteristics of the six included RCTs are listed in S1 Table in the supporting information; these RCTs included a total study population of 2102 randomized participants (sample sizes ranging from 76 [32] to 700 patients [31]). The process of data extraction had a kappa value of 0.92, demonstrating high agreement between the two authors. Three studies [27, 28, 31] were multicenter trials. The results of these trials were published during or after 2015. All studies enrolled participants aged 65 years or older. All studies were randomized placebo-controlled trials. In all included studies, the diagnosis of delirium was based on the confusion assessment method (CAM) or CAM for the intensive care unit (CAM-ICU). Both the CAM and CAM-ICU are well-established delirium assessment instruments that have been widely used in clinical trials [1, 9, 33, 34]. In the included trials, the types of noncardiac surgery varied and included intra-abdominal, intra-thoracic, spinal and extremital, orthopedic, superficial, and urologic surgery. Additionally, the intervention time of dexmedetomidine was different among the included studies. In three studies [27, 29, 30], dexmedetomidine was used during the intraoperative period. In two trials [31, 32], dexmedetomidine was administered after surgery. In another study [28], dexmedetomidine was administered in both the intraoperative and postoperative periods.

### Quality assessment

**Fig 2:**
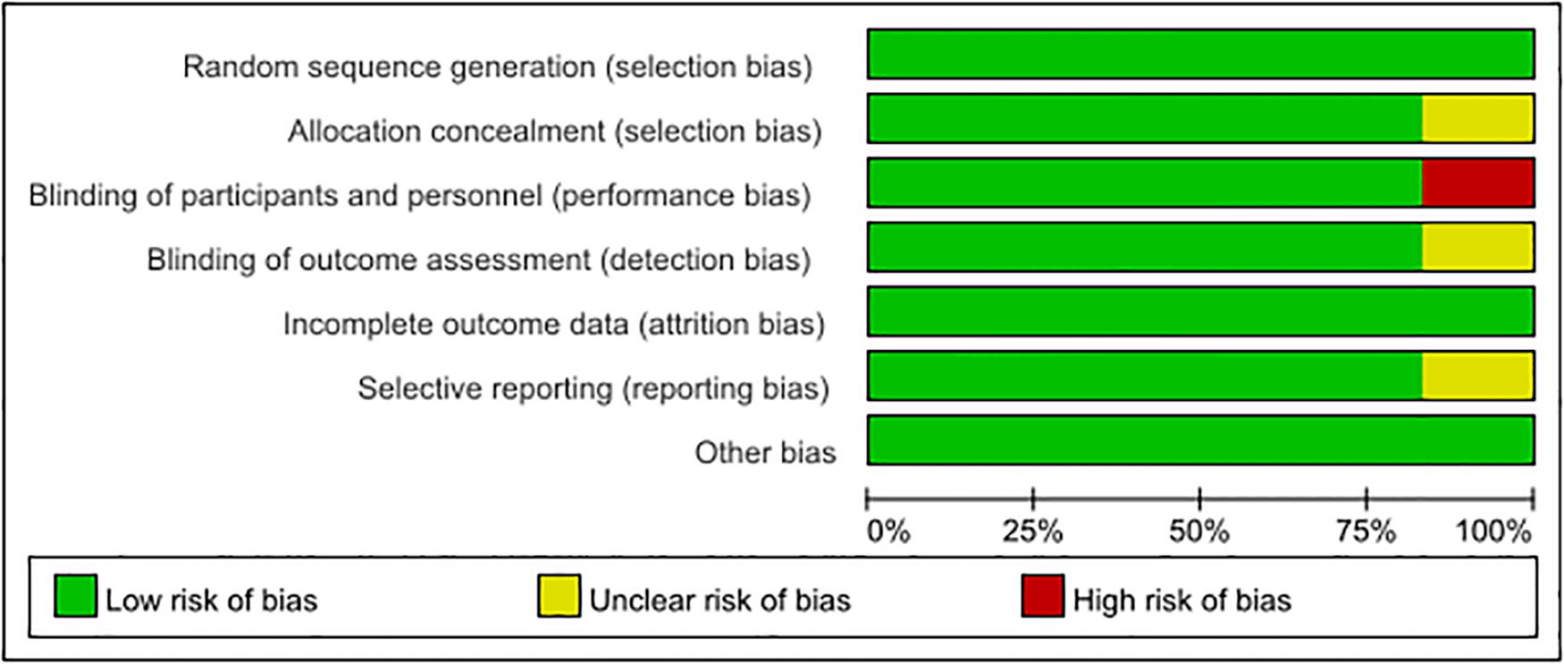
Risk of bias graph.

The results of the methodological quality assessment are summarized in the Fig 2. Following strict judgments of each included study according to the Cochrane handbook, three trials [28, 31, 32] were assessed as high quality as a result of having low risks of methodological bias across all seven domains. One study [29] was considered low quality; there was a high risk of bias in the blinding of the researchers, because the anesthesiologists and nurses were not blinded to anesthetic agents. In one study [27], there was inadequate information to decide whether a risk of selective reporting bias existed; thus, this study was considered as a moderate-quality study. In addition, another study [30] was considered to be of moderate quality due to an unclear risk of bias as a consequence of inadequate information in both assignment concealment and blinding of the outcome evaluation.

### Results of the meta-analysis

#### POD

**Fig 3:**
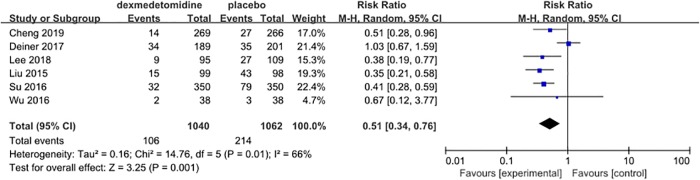
Meta-analysis of POD.

The incidence of POD was reported in six studies [27–32], with 320 events (15.2%) among 2102 patients. The reported incidence of POD among the included studies ranged from 6.6% [32] to 29.4% [30]. The overall pooled crude incidence of POD differed significantly between the dexmedetomidine and placebo groups. Only 106 of the 1040 patients in the dexmedetomidine group were reported to have POD, which accounted for 10.2% of the patients, while 214 of the 1062 patients in the placebo group were reported to have POD, which was a rate of 20.2%. The Pooled data from the six trials revealed that dexmedetomidine could significantly decrease the incidence of POD compared with placebo (RR = 0.61, 95% CI 0.34–0.76, *P* = 0.001, *I*^*2*^ = 66%; Fig 3).

#### Bradycardia

**Fig 4:**
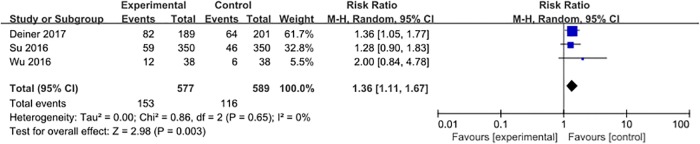
Meta-analysis of bradycardia.

Three studies [28, 31, 32] reported the incidence of bradycardia, with 269 events (23.1%) among 1166 patients. Dexmedetomidine, compared to placebo, was associated with a significant increase in the risk of perioperative bradycardia (RR = 1.36, 95% CI 1.11–1.67, *P* = 0.003, *I*^*2*^ = 0%; Fig 4).

#### Hypotension

**Fig 5:**
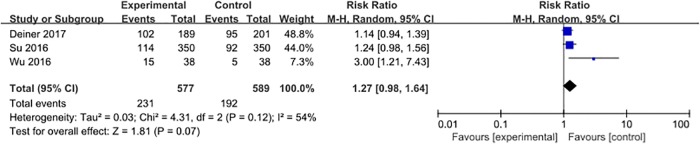
Meta-analysis of hypotension.

Three studies [28, 31, 32] reported the occurrence of hypotension, with 423 events (36.3%) among 1166 patients. Dexmedetomidine had no significant influence on the occurrence of perioperative hypotension when compared with placebo (RR = 1.27, 95% CI 0.98–1.64, *P* = 0.07, *I*^*2*^ = 54%; Fig 5).

#### Hypertension

**Fig 6:**
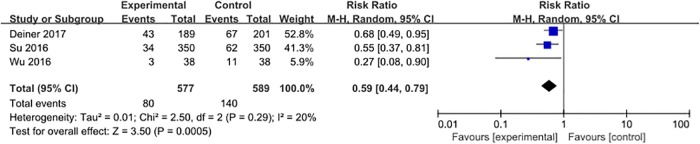
Meta-analysis of hypertension.

Three studies [28, 31, 32] reported the incidence of hypertension, with 220 events (18.7%) among 1166 patients. The pooled analysis demonstrated that dexmedetomidine, compared to placebo, could significantly decrease the incidence of perioperative hypertension (RR = 0.59, 95% CI 0.44–0.79, *P <* 0.001, *I*^*2*^ = 20%; Fig 6).

#### Tachycardia

**Fig 7:**
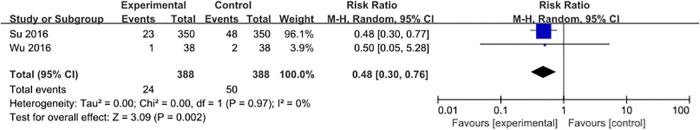
Meta-analysis of tachycardia.

Two studies [31, 32] reported the occurrence of tachycardia, with 74 events (9.5%) among 776 patients. The pooled analysis demonstrated that dexmedetomidine, compared to placebo, was associated with a statistically significant decrease in the prevalence of perioperative tachycardia (RR = 0.48, 95% CI 0.30–0.76, *P* = 0.002, *I*^*2*^ = 0%; Fig 7).

#### Myocardial infarction

**Fig 8:**
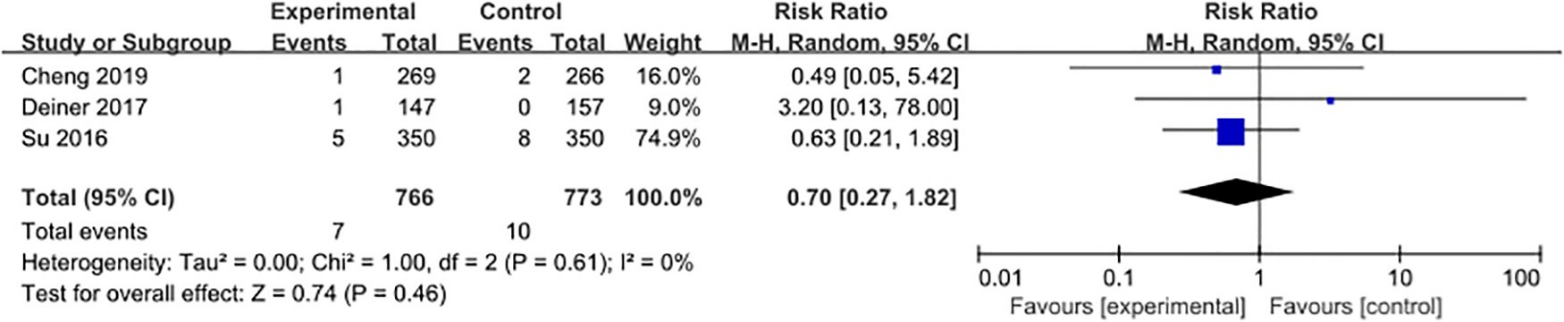
Meta-analysis of myocardial infarction.

Three studies [27, 28, 31] reported the occurrence of myocardial infarction, with 17 events (1.1%) among 1539 patients. The pooled data from these studies showed that dexmedetomidine had no significant influence on the incidence of myocardial infarction when compared with placebo (RR = 0.70, 95% CI 0.27–1.82, *P* = 0.46, *I*^*2*^ = 0%; Fig 8).

#### Stroke

**Fig 9:**
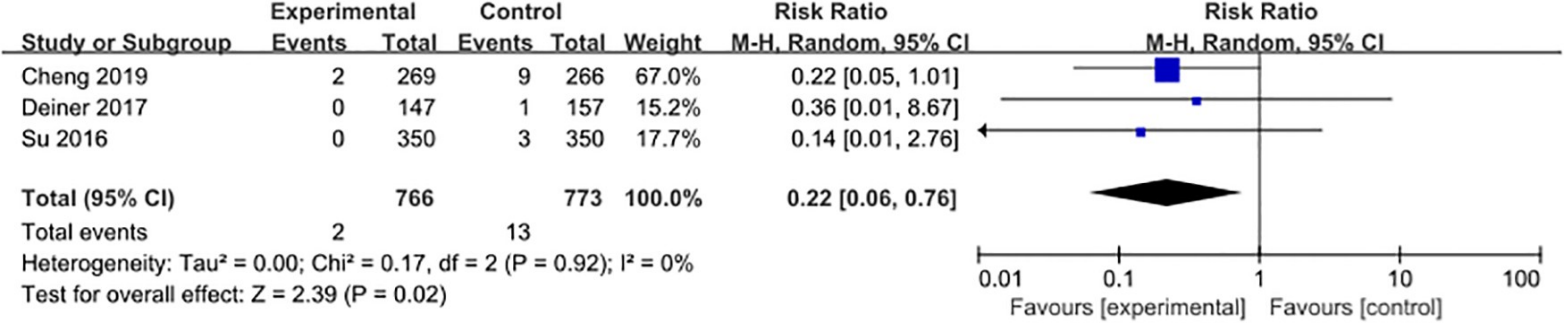
Meta-analysis of stroke.

Three studies [27, 28, 31] reported the incidence of stroke, with 15 events (1.0%) among 1539 patients. The pooled results suggested that the use of dexmedetomidine might be associated with a reduced incidence of stroke when compared with placebo (RR = 0.22, 95% CI 0.06–0.76, *P* = 0.02, *I*^*2*^ = 0%; Fig 9).

#### Hypoxaemia

**Fig 10:**
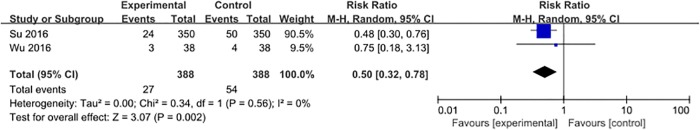
Meta-analysis of hypoxaemia.

Two studies [31, 32] reported the incidence of hypoxaemia, with 81 events (10.4%) among 776 patients. The pooled data from three studies showed that dexmedetomidine had no significant influence on the occurrence of hypoxaemia when compared with placebo (RR = 0.50, 95% CI 0.32–0.78, *P* = 0.002, *I*^*2*^ = 0%; Fig 10).

#### All-cause mortality

**Fig 11:**
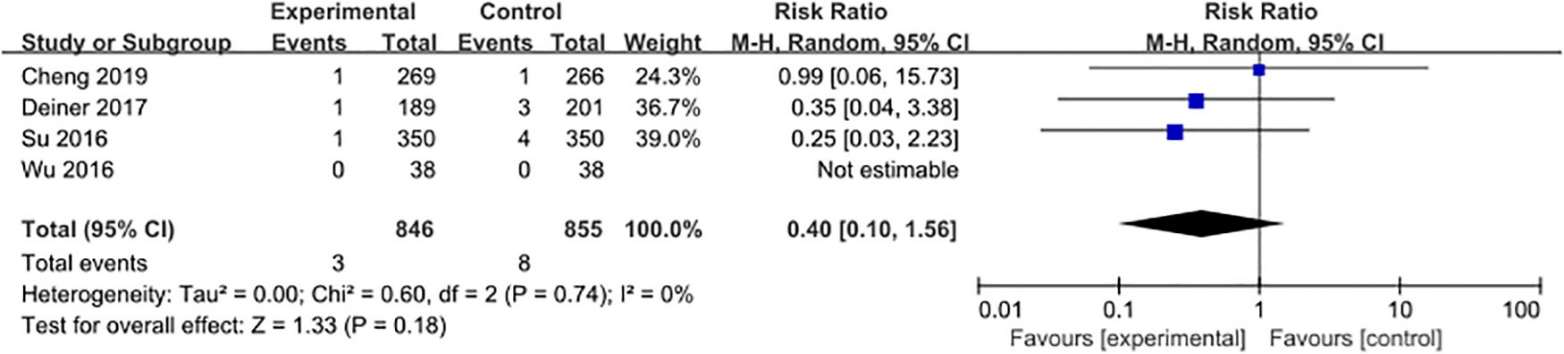
Meta-analysis of all-cause mortality.

Four studies [27, 28, 31, 32] reported the incidence of all-cause mortality, including 11 events (0.6%) among a total of 1701 patients. The pooled analysis showed that dexmedetomidine did not cause a significant decrease in the risk of all-cause mortality when compared with placebo (RR = 0.40, 95% CI 0.10–1.56, *P* = 0.18, *I*^*2*^ = 0%; Fig 11).

### Publication bias

**Fig 12:**
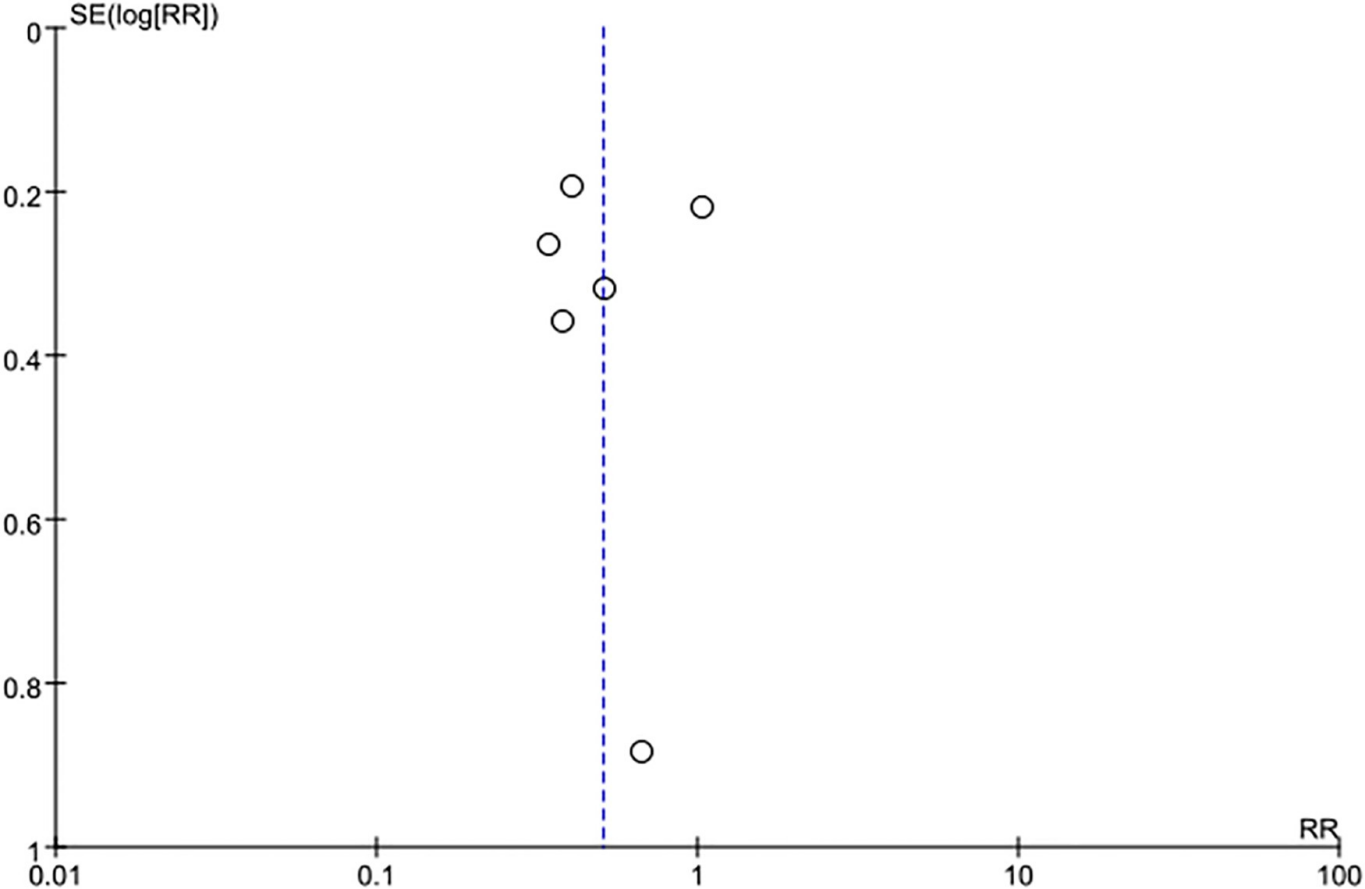
Funnel plot of POD.

Using the incidence of POD as the primary outcome, a visual inspection of the funnel plot (Fig 12) showed no apparent evidence of publication bias. Furthermore, no obvious evidence of publication bias was identified, with an Egger’s test P value of 0.89 and a Begg’s rank correlation of 0.71.

## Discussion

The meta-analysis incorporated six RCTs [27–32] that met the inclusion criteria and included 2102 elderly patients in total. The main conclusion of this review was that elderly patients who underwent noncardiac surgery and received dexmedetomidine intervention had a significantly lower occurrence of POD than those who received placebo. Even though dexmedetomidine could increase the risk of bradycardia, the drug might reduce the occurrence of tachycardia, hypertension, stroke, and hypoxaemia; no significant differences were discovered in the incidence of hypotension, myocardial infarction, and all-cause mortality between the dexmedetomidine and placebo groups.

Postoperative delirium is an acute cognitive disturbance that plays an important role in postoperative outcomes and remains a serious and costly condition, particularly in elderly patients [35]. The etiology of delirium is multifactorial (e.g., history of dementia or cognitive impairment and old age), the prominent potential pathophysiologic contributors of developing delirium involve abnormal levels of neurotransmitters and pro-inflammatory markers, physiologic stressors, metabolic derangements, and electrolyte disorders plus genetic factors [1]. There might be an interplay between the inflammatory response of the peripheral innate immune system and the human brain that is potentially associated with cognitive function impairments after surgery with general anesthesia [36].

Dexmedetomidine, a highly selective alpha-2 adrenoreceptor agonist, has the positive sedation, anti-anxiety, and analgesic effects [12, 13, 37]. Thus, dexmedetomidine has been widely used in surgical patients. Because of the complex etiology and pathophysiology of delirium, the accurate mechanisms by which dexmedetomidine could decrease the risk of delirium remain poorly understood, although there are some studies on this issue. Dexmedetomidine could suppress systemic inflammatory processes through the downregulation of the HMGB1-TLR4-NF-κB signaling pathway by activating alpha-2 adrenergic receptors and stimulating the vagus nerve via a vagal- and alpha-7 nicotinic acetylcholine receptor-dependent mechanism [38, 39]. Dexmedetomidine might confer protective effects against transient cerebral ischemia or ischemic reperfusion impairment via restraining inflammation and anesthesia-induced (e.g., propofol) neurotoxicity in the brain [12, 40]. The findings of these studies are consistent with the results of our meta-analysis, which suggest that perioperative intervention with dexmedetomidine might be a promising option for ameliorating the occurrence of delirium following surgery with general anesthesia.

Previous reviews [15, 16] demonstrated that the perioperative usage of dexmedetomidine was related to a reduced prevalence of delirium in adult patients who underwent cardiac surgery. In addition, Duan et al. [41] assessed the efficacy of dexmedetomidine for delirium prevention among adult patients after noncardiac surgery and revealed similar results. However, these studies [15, 16, 41] compared dexmedetomidine with other sedative drugs (e.g., midazolam and propofol) that activate γ-aminobutyric acid A (GABA_A_) receptors. Notably, the current evidence indicates that the use of GABA_A_ agonists is associated with a high risk of developing delirium [9, 11, 42–45]. One plausible interpretation is that dexmedetomidine does not induce delirium as do these GABA_A_ agonists, but also does not prevent delirium [31]. To clearly illuminate this controversial topic, we conducted this meta-analysis and confirmed that dexmedetomidine could effectively prevent POD among elderly patients after noncardiac surgery when compared with placebo.

Among the included studies, four RCTs [27, 29–31] found that dexmedetomidine treatment could reduce the incidence of delirium while the results observed in the remaining studies [28, 32] were in contrast with these findings. In the study by Su and his colleagues [31], patients who received a continuous intravenous infusion of dexmedetomidine from study recruitment on the day of surgery until postoperative day 1 were observed to have a decreased risk of delirium. However, Deiner and his colleagues [28] limited dexmedetomidine administration to intraoperative period and 2 hours into recovery. The lack of salutary effects might be partly due to the short-acting nature of the drug and the unsuitable timing of drug infusion. In addition, the incidence of delirium was not the primary endpoint of the relatively small study by Wu et al [32], which had a sample size of 61 patients.

The most common adverse effects of dexmedetomidine that have been reported are bradycardia and hypotension, which are the adverse consequences of stimulating alpha-2 adrenoreceptors [46, 47]. Dexmedetomidine is an excellent drug that could not only reduce the magnitude of the hemodynamic response to anesthetic induction, tracheal intubation, surgery and extubation, but could also decrease the consumption of opioids and isoflurane to achieve appropriate analgesia and anesthesia [48, 49]. Our study also discovered that dexmedetomidine could reduce the risk of tachycardia and hypertension in elderly patients who underwent noncardiac surgery, but dexmedetomidine might increase the incidence of perioperative bradycardia; these findings are in accordance with the results of two previous meta-analyses [15, 16]. Notably, these adverse effects are both multifactorial and dosage-dependent and are mediated via both central and peripheral mechanisms [47].

Myocardial infarction and stroke are common cardiovascular complications following surgery and are strongly related to a high risk of mortality [50, 51]. Our meta-analysis did not observe any significant differences between the dexmedetomidine and placebo groups in the occurrence of myocardial infarction and all-cause mortality. A prior Cochrane review [52] revealed similar conclusions. Moreover, as observed in our study, the usage of dexmedetomidine in the perioperative period might be associated with a reduced incidence of stroke, but more large-scale cardiovascular outcome trials are required to confirm this finding. In addition, dexmedetomidine was related to a decreased risk of hypoxaemia, which indicated that dexmedetomidine might not result in respiratory depression. This result is in line with another study [53] that showed that dexmedetomidine had no harmful clinical effects on respiration when used in the surgical population that required intensive care.

However, this review has several limitations. First, some restrictions exist in number and quality of the included trials, since only six RCTs were included in our study. A high risk of bias in the blinding of researchers was identified in one study [29], and two other studies [27, 30] were considered moderate quality in consequence of unclear risk from inadequate descriptions of the allocation concealment process, blinding of outcome assessments or selective reporting. Second, we identified moderate heterogeneity among the included trials, including in variables such as intervention time (intraoperative, intraoperative plus postoperative, and postoperative), type of surgery, sedative dose and rate of dexmedetomidine infusion, and patient characteristics. Moreover, there was an inadequate number of trials to apply meta-regression methods that could assess the variables potentially related to heterogeneity. Finally, although no statistical evidence of publication bias was observed, the probability of bias still exists as a consequence of the low statistical power caused by the limited quantity of included studies.

Despite the limitations above, to the best of our knowledge, this review was the first meta-analysis to estimate the prophylaxis efficacy of dexmedetomidine on POD when compared with placebo in elderly patients after noncardiac surgery. Furthermore, our review only included randomized double-blind placebo-controlled clinical trials, whose rigorous study designs strengthens the creditability of the main outcomes.

## Conclusion

In summary, the results of our review indicated that perioperative prophylactic intervention with dexmedetomidine, compared with placebo, could significantly reduce the prevalence of POD, tachycardia, hypertension, and hypoxaemia in elderly patients following non-cardiac surgery. However, the use of dexmedetomidine was associated with an elevated risk of bradycardia. Additional high-quality, large-scale multicenter RCTs are still warranted for the purpose of exploring the optimal dose and timing of dexmedetomidine on POD prevention among the elderly population after noncardiac surgery.

## Supporting information

S1 Checklist PRISMA Checklist. (DOC)

S1 File Search strategies. (DOC)

S1 Table The characteristics of included studies. (DOC)
