# Supplementary material for: Dexmedetomidine for the prevention of postoperative delirium in elderly patients undergoing noncardiac surgery: A meta-analysis of randomized controlled trials
Source: PLoS One. 2019 Aug 16;14(8):e0218088. doi: 10.1371/journal.pone.0218088 (PMC6697366; doi:10.1371/journal.pone.0218088)
Supplement: S1 File — (DOC) [file pone.0218088.s002.doc]

Search strategy in PubMed.

#1.

dexmedetomidine [mesh] OR dexmedetomidine [tiab] OR Precedex [tiab]

#2.

"Saline Solution"[mesh] OR Placebos[mesh] OR placebo[tiab] OR "Sodium Chloride"[mesh] OR saline[tiab] OR "normal saline "[tiab] OR "salt water"[tiab]

#3.

aged[mesh] OR elderly[tiab] OR "frail elderly"[mesh] OR old*[tiab] OR geriatric*[tiab] OR "aged 80 and over "[tiab]

#4.

intraoperative[tiab] OR operation[tiab] OR surgery[tiab] OR surgical[tiab] OR postoperative[tiab] OR anesthesia [mesh] OR anaesthesia[tiab] OR operative[tiab] OR perioperative[tiab]

#5.

"randomized controlled trial"[pt] OR "controlled clinical trial"[pt] OR randomized[tiab] OR placebo[tiab] OR "drug therapy"[sh] OR randomly[tiab] OR trial[tiab] OR groups[tiab]

#6. #1 AND #2 AND #3 AND #4 AND #5

Search strategy in Embase.

#1.

'dexmedetomidine':ab,ti OR 'Precedex*':ab,ti

#2.

'Saline Solution':ab,ti OR 'Placebos':ab,ti OR 'placebo':ab,ti OR 'Sodium Chloride*':ab,ti OR 'saline*':ab,ti OR 'normal saline':ab,ti OR 'salt water':ab,ti

#3.

'aged':ab,ti OR 'elderly':ab,ti OR 'frail elderly':ab,ti OR 'old*':ab,ti OR 'geriatric*':ab,ti OR 'aged 80 and over':ab,ti

#4.

'surgery':ab,ti OR 'surgical':ab,ti OR 'operation':ab,ti OR 'postoperative':ab,ti OR 'anesthesiological':ab,ti OR 'anaesthesia':ab,ti OR 'anesthesia':ab,ti OR 'perioperative':ab,ti OR 'intraoperative':ab,ti

#5.

('clinical':ti,ab AND 'trial':ti,ab) OR 'clinical trial'/exp OR random* OR 'drug therapy':lnk

#6. #1 AND #2 AND #3 AND #4 AND #5
