# Supplementary material for: Dexmedetomidine for the prevention of postoperative delirium in elderly patients undergoing noncardiac surgery: A meta-analysis of randomized controlled trials
Source: PLoS One. 2019 Aug 16;14(8):e0218088. doi: 10.1371/journal.pone.0218088 (PMC6697366; doi:10.1371/journal.pone.0218088)
Supplement: S1 Table — (DOC) [file pone.0218088.s003.doc]

| **Study** | **Intervention versus control** | **Gender (M/F)** | **Age (y) (Mean±SD)** | **Evaluation time(d)** | **POD Incidence (n/total) (%)** | **Outcomes** |
| --- | --- | --- | --- | --- | --- | --- |
| Cheng et al, 2019 (China) | DEX vs placebo | C:194/75 I:198/68 | C:71.0  (67.0-75.0) I:70.0  (68.0-74.0) | time in the recovery area | C:14/269(5.2) I:27/266(10.2) | POD,  MI,stroke,  mortality |
| Deiner  et al, 2017  (United States) | DEX vs placebo | C:98/103 I:92/97 | C:74.0  (71.0-78.0) I:74.0  (71.0-78.0) | Day 1-5 | C:23/201(11.4) I:23/189(12.2) | POD, bradycardia, hypotension, hypertension,  MI,stroke,  mortality |
| Lee et al, 2018 ( Korea) | DEX vs placebo | C:47/62 I:44/51 | C:73.8±6.1 I:72.2±5.4 | Day 1-5 | C:27/109(24.8) I:9/95(9.5) | POD |
| Liu et al, 2015 (China) | DEX vs placebo | C_1_:29/29  C_2_:23/17 I_1_:26/34 I_2_:18/21 | C_1_:72.8±9.2  C_1_:75.3±7.8 I_2_:71.2±8.1 I_2_:72.8±8.2 | Day 1,3,5 | C_1_:18/58(31.0)  C_2_:25/40(62.5) I_1_:5/60(8.3) I_2_:10/39(25.6) | POD |
| Su et al, 2016 (China) | DEX vs placebo | C:202/148 I：221/129 | C:74.4±7.0 I:74.3±6.7 | Day 1-7 | C:79/350(23) I:32/350(9) | POD, bradycardia, hypotension, hypertension,  tarchycardia,  MI,stroke,  hypoxaemia,  mortality |
| Wu et al, 2016 (China) | DEX vs placebo | C: 20/18 I：24/18 | C:76±6 I:74±5 | Day 1-7 | C:3/38(7.9) I:2/38(5.3) | POD, bradycardia, hypotension, hypertension,  tarchycardia,  hypoxaemia,  mortality |

**S1 Table.** **Characteristics of included studies**.

**Table 1.** **Characteristics of included studies** (Continued).

| **Study** | **Surgery type** | **Definition delirium** | **Type of Anaesthesia** | **Risk of bias** | **DEX intervention time** | **the sedative dose and rate of DEX infusion** |
| --- | --- | --- | --- | --- | --- | --- |
| Cheng et al, 2019 (China) | gastro-intestinal laparotomy | CAM-ICU | GA | unclear | induction of anesthesia～30 min before the end of surgery | 0.5μg/kg bolus followed by 0.4μg/kg/h |
| Deiner  et al, 2017  (United States) | non-  cardiac | CAM/ CAM-ICU | GA | low | entering the operating room～2 h into recovery | 0.5μg/kg/h |
| Lee et al, 2018 (South Korea) | non-  cardiac | CAM | GA | high | induction of anesthesia～the end of surgery | 1μg/kg bolus followed by 0.2-0.7μg/kg/h |
| Liu et al, 2015 (China) | joint replacement | CAM | GA | unclear | throughout the duration of the surgery | 0.2-0.4μg/kg/h |
| Su et al, 2016 (China) | non-  cardiac | CAM-ICU | GA | low | intensive care unit admission～8：00 postoperative day 1 | 0.1μg/kg/h |
| Wu et al, 2016 (China) | non-  cardiac | CAM | GA | low | 15 h, i.e., from 5:00 PM on the day of surgery until 8:00 AM on the first day after surgery | 0.1ug/kg/h |

The values are showed as the mean (standard deviation), number (percentage, %) or median (interquartile range).

I, intervention; C, control; DEX, dexmedetomidine; Evaluation time, postoperative day; POD, postoperative delirium; MI, myocardial infaction; GA, general anaesthesia; CAM, confusion assessment method; CAM-ICU, CAM for the intensive care unit.
